# New Approaches for Improving the Quality of Processed Fruits and Vegetables and Their By-Products

**DOI:** 10.3390/foods12071353

**Published:** 2023-03-23

**Authors:** Fátima A. Miller, Teresa R. S. Brandão, Cristina L. M. Silva

**Affiliations:** Universidade Católica Portuguesa, CBQF—Centro de Biotecnologia e Química Fina—Laboratório Associado, Escola Superior de Biotecnologia, Rua Diogo Botelho 1327, 4169-005 Porto, Portugal; tbrandao@ucp.pt (T.R.S.B.); clsilva@ucp.pt (C.L.M.S.)

The 2030 Sustainable Development Agenda calls for all social actors to contribute to significant societal and environmental issues. Food scientists and professionals have a fundamental role to play in increasing the availability of food and minimizing the environmental impact. Appropriate actions include a reduction in food losses and waste, minimizing water and energy consumption, adding value and using by-products in innovative solutions, the use of new or improved processing technologies to ensure safety and maximum quality, optimizing processes and distribution conditions, and producing enough food with health benefits for the consumer.

Fruits and vegetables consumption is essential to a healthy diet, reducing the risk of cancer, diabetes, and heart disease. They are a source of vitamins, minerals, phytochemicals, and fiber. Parts of the fruits that are usually not consumed, such as peel, seeds, and pomaces, are also rich in these compounds. Strategies to transform and include them in the food chain should be considered a global approach to achieving sustainable development goals (SDGs). Moreover, new products, processes, and distribution conditions should contribute to increasing fruit and vegetable consumption, improving the quality of products, and minimizing energy and water consumption, as well as losses and waste.

This Special Issue includes 15 papers (4 reviews) highlighting the most significant scientific contributions and progress in this field.

It is of general knowledge that fresh produce can be microbiologically contaminated throughout the supply chain, from production, processing, transporting, storage, and sale sites to our kitchen benches. Although consumers demand minimally processed or “fresh-like” food products, adequate processing must be ensured to guarantee product safety. Pre-treatments have been widely used in different fruits and vegetables to improve their stability for consumption and commercialization and extend the product’s shelf-life. An example of this is the study of Tavares et al. [1], where the blanching of fresh cowpeas contributed to enzymatic inactivation and microbiological control, promoting product conservation. The results showed that the ideal blanching conditions are 70 °C for 4 min, where significant quality losses are minimized, and the shelf-life of the samples is extended by 5 days.

Many foodborne outbreaks are associated with the consumption of fruits and vegetables. In light of this knowledge, extensive research efforts are being made to find feasible technologies that guarantee microbial pathogen inactivation while maintaining product quality. Two of the most common pathogenic bacteria isolated from fresh produce are *Staphylococcus aureus* and *Listeria monocytogenes*. Hence, Kim et al. [2] explored the effect of combined hurdle technologies (slightly acidic electrolyzed water—SAEW, fumaric acid—FA, and UV-C waterproof light-emitting diodes—UVC W-LED) for the control of these two microorganisms in fresh-cut fruits. The results showed that combining the three treatments decreased the microbial load (0.51 to 2.63 log CFU/g) more efficiently in grapes, fresh-cut apples, cherry tomatoes, and pineapples compared to single treatments or other combinations.

*Alicyclobacillus acidoterrestris* is a fruit juice spoiler that produces undesirable off-flavors which can survive after thermal processing, even in acidic conditions. Therefore, this microorganism has been proposed as a safety target to control the effectiveness of juices’ heat treatments. In their review, Sourri et al. [3] discussed several control and prevention methods to inactivate this hazardous bacterium to obtain safe fruit juices with an extended shelf-life. Chemical (oxidants or natural compounds), physical (thermal pasteurization), and non-thermal (high hydrostatic pressure, high-pressure homogenization, ultrasound, UV-C light, irradiation, microwaves, ohmic heating, and pulse electric field) treatments were revised. The authors also highlighted the importance of improving the existing techniques for the rapid and early detection of *A. acidoterrestris*.

Vitamin C is a natural antioxidant present in many fruits and vegetables. Due to its heat sensitivity and water-soluble characteristics, significant losses of this compound are reported to occur, especially after conventional thermal treatments. For these reasons, novel technologies are being studied to achieve a final product with fewer losses in vitamin C content. As this compound is currently used as a food quality indicator, Giannakourou and Taoukis [4] published a review addressing the vitamin C degradation pathways, the effect of conventional and novel preservation processes on vitamin C retention, and its degradation kinetics. This critical overview emphasizes the importance of including more sophisticated mathematical and statistical methodologies integrating parameter uncertainties to provide more realistic predictions and better control of the food processes.

Another antioxidant naturally occurring in foods is tocochromanol, which belongs to vitamin E molecules and plays a role in cell and tissue protection. In this context, Zhao et al. [5] studied the changes in tocochromanol accumulation in pomelo fruit at different development stages. The results demonstrated a relationship between gene transcription levels and tocochromanol contents in young pomelo. Therefore, the best extraction period can be identified, and young pomelo fruit can have high additional value utilization.

The application of novel food packaging methodologies (edible films and coatings) has increased in recent decades due to the need to develop sustainable preservation techniques. Mitelu et al.’s review [6] collected the latest research on edible films and coatings, their composition and application methods, and their effect on food quality and shelf-life. This review also provides the state of the art of edible films and coatings with functional additives for different minimally processed fruits and vegetables. As stated by the authors, the most recent developments are characterized by the extensive use of chitosan or alginate as the main component of the edible coatings to which different functional ingredients are added, such as essential oils (EOs), nano-forms, antioxidants extracts, and probiotics. This is the case in Tampucci et al.’s [7] work, where tyrosol was introduced into tomato fruit by applying a chitosan–tyrosol coating. In vivo and in vitro permeation tests revealed tyrosol’s ability to permeate across the fruit peel and to be present for up to 7 days of storage. Therefore, the authors proved the chitosan coating’s effectiveness in producing functional food products under room temperature conditions. Research to date is incredibly encouraging in this field, and the further investigation of consumers’ acceptance of these novel packaging techniques is recommended.

Considerable research has been conducted on mild processing techniques. The overview conducted by Soni and Brightwell [8] outlines the recent applications of thermal and hurdle approaches to enhance food safety and its effects on fruit’s and vegetable’s sensory and nutritional qualities. This review discusses alternative technologies involving moderate thermal treatment, such as pulsed electric fields, pressure-assisted thermal processing, and microwave-assisted thermal sterilization. Although these novel technologies need more research at temperatures below 100 °C, the authors recognize their potential for industrial applications and provide advice for studies on their social, economic, and financial impact.

High-pressure processes are being extensively investigated with promising results. High-pressure homogenization (HPH) has been used for microbial inactivation, emulsions preparation, particle size reductions, and as a lever for improving food products’ rheological properties and shelf-life extension. More recently, this technology has been applied to the extraction of bioactive compounds based on the distribution of shear stress across a product, increasing the functionality of these compounds. This is the case in Gottardi et al.’s [9] study, which examined the combined effect of HPH and fermentation using a lactic acid bacterium as the biocontrol agent of carrot juice. The results confirmed an improvement in the juice functionality since an increase in the β-carotene and lutein content was observed, and their stability was retained over time.

Another well-known preservation method used on many food products is high-pressure processing (HPP). This technique guarantees food safety and increases the shelf-life while maintaining the quality attributes of fresh products. In the work of Li and Padilla-Zakour [10], they compared the whole Concord grape puree quality during its refrigerated shelf-life by pre-applying HPP or pasteurization. Their research proved that HPP-treated samples had a better consumer acceptability due to a more fresh-like and nutritious feature than the pasteurized ones. It was also suggested that HPP can improve bioactive compound extractability due to changes in the food structure of the product. In this context, Lara-Abia et al. [11] evaluated the impact of HPP on extracting individual carotenoid and carotenoid ester from papaya and assessed their bioaccessibility using an in vitro simulated gastrointestinal digestion assay. The results showed a high carotenoid stability but very low bioaccessibility. Consequently, these authors expect to perform further studies soon to improve these compounds’ bioaccessibility. Another approach for recovering bioactive compounds from strawberry pomace was detailed by Pukalskienè et al. [12]. They compared conventional solid–liquid and pressurized liquid extraction methods and assessed the bioactivity of these compounds in human cell cultures. In general, pressurized liquid extracts demonstrated solid antioxidant potential and may be considered promising antiproliferative substances in disease prevention.

It is well documented that fruit by-products, usually discarded and not consumed, are abundant sources of bioactive compounds and nutrients with health benefit properties. Since industrial by-products are rich in organic nature, they represent one of the earth’s most extensive renewable resources. Therefore, it is of great interest, both from economic and environmental points of view, to reuse these wastes. In this framework, two papers assessed the impact of two different preservation processes on melon peel’s physicochemical, nutritional, and microbiological indicators. The paper of Miller et al. [13] showed that melon peel cubes retained their main quality characteristics after a gaseous ozone treatment of 30 min. The application of this process also allowed for an inactivation of 1.23 ± 0.10 log cfu/mL of *L. innocua*, used as a surrogate of the pathogenic *L. monocytogenes*. When freeze-drying was used, as in the work of Sroy et al. [14], the authors came to similar conclusions. An ozone pre-treatment led to dried melon samples with a better nutritional quality. Both studies agreed that although raw melon peel is a non-edible by-product, it can be a value-added ingredient if conveniently processed and transformed.

This Special Issue also includes one paper on the water–energy–food nexus. Fernández-Ríos et al.’s work [15] aimed to understand and assess potato chips’ environmental impacts and nutritional quality by applying the life cycle assessment tool. The results showed that the cultivation and processing phases significantly contribute to environmental problems, which should improve the implementation of sustainable natural resources and low-impact technologies. The study also suggests relying on a circular economy to promote sustainability within the context of the globalization of the food sector.

In summary, the papers published in this Special Issue discuss the current and future challenges of advanced processing technologies applied to fruits, vegetables, and their by-products. A common main goal was to attain high-quality food products that met consumers’ demand for freshness and convenience without compromising the safe microbiological standards for consumption. The papers also highlight the importance of involving all the intervenient aspects of the food supply chain to mitigate the environmental impact by applying more sustainable and environmentally friendly solutions.
